# Supplementary figures and images for: BLOC1S1 regulates autolysosomal and exosomal dynamics during CD4+ T cell differentiation
Source: bioRxiv. 2026 May 18:2026.05.14.725149. Preprint. [Version 1] doi: 10.64898/2026.05.14.725149 (PMC13228349; doi:10.64898/2026.05.14.725149)

Supplementary Figure 1

A

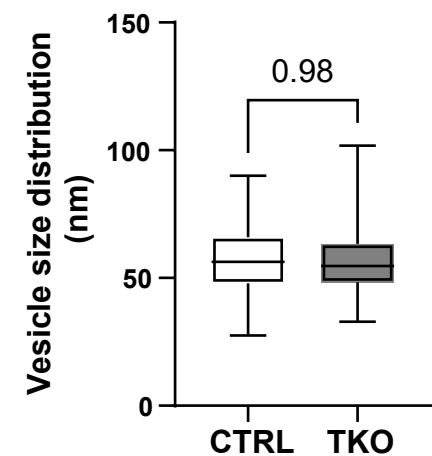

B

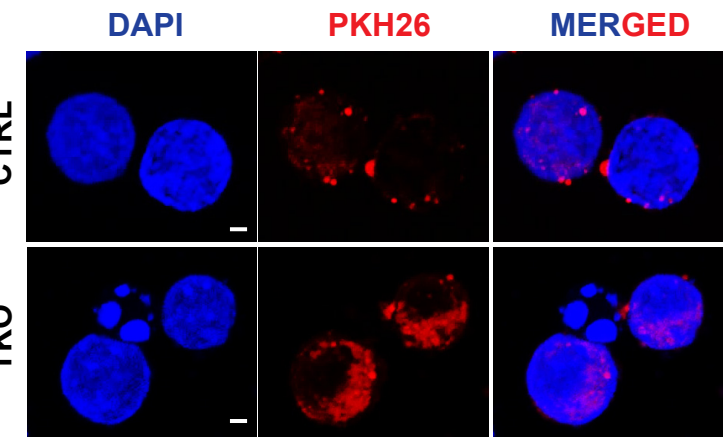

C

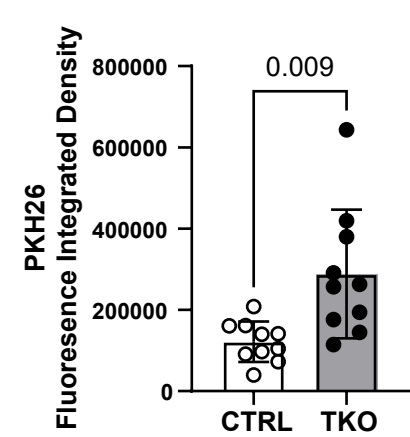

D

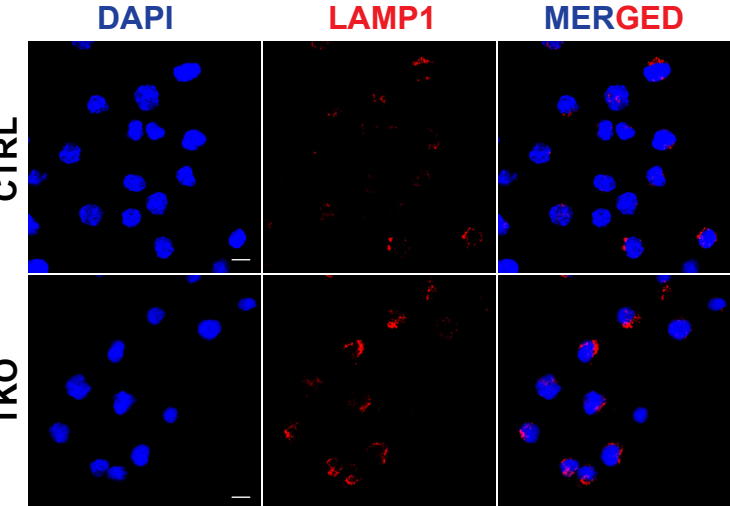

E

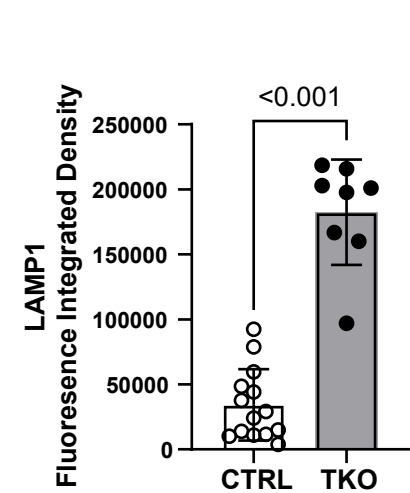

Supplement: Supplement 1 — Supplementary Figure 1. Increased PKH26 and LAMP1 accumulation in BLOC1S1 deficient CD4+ T cells. (A) Quantification of vesicle size distribution measured from TEM images. (B) Representative confocal microscopy images of CD4+ T cells stained with PKH26 (red) and DAPI (blue). Scale bars, 2 μm. (C) Quantification of PKH26 fluorescence integrated density. (D) Representative confocal microscopy images of CD4+ T cells stained with anti-LAMP1 antibody (red) and DAPI (blue). Scale bars, 5 μm. (E) Quantification of LAMP1 fluorescence integrated density. Data are presented as dot plots with mean ± SEM. Statistical analysis was performed using an unpaired two-tailed Student’s t-test. [file media-1.pdf]

Supplementary Figure 4

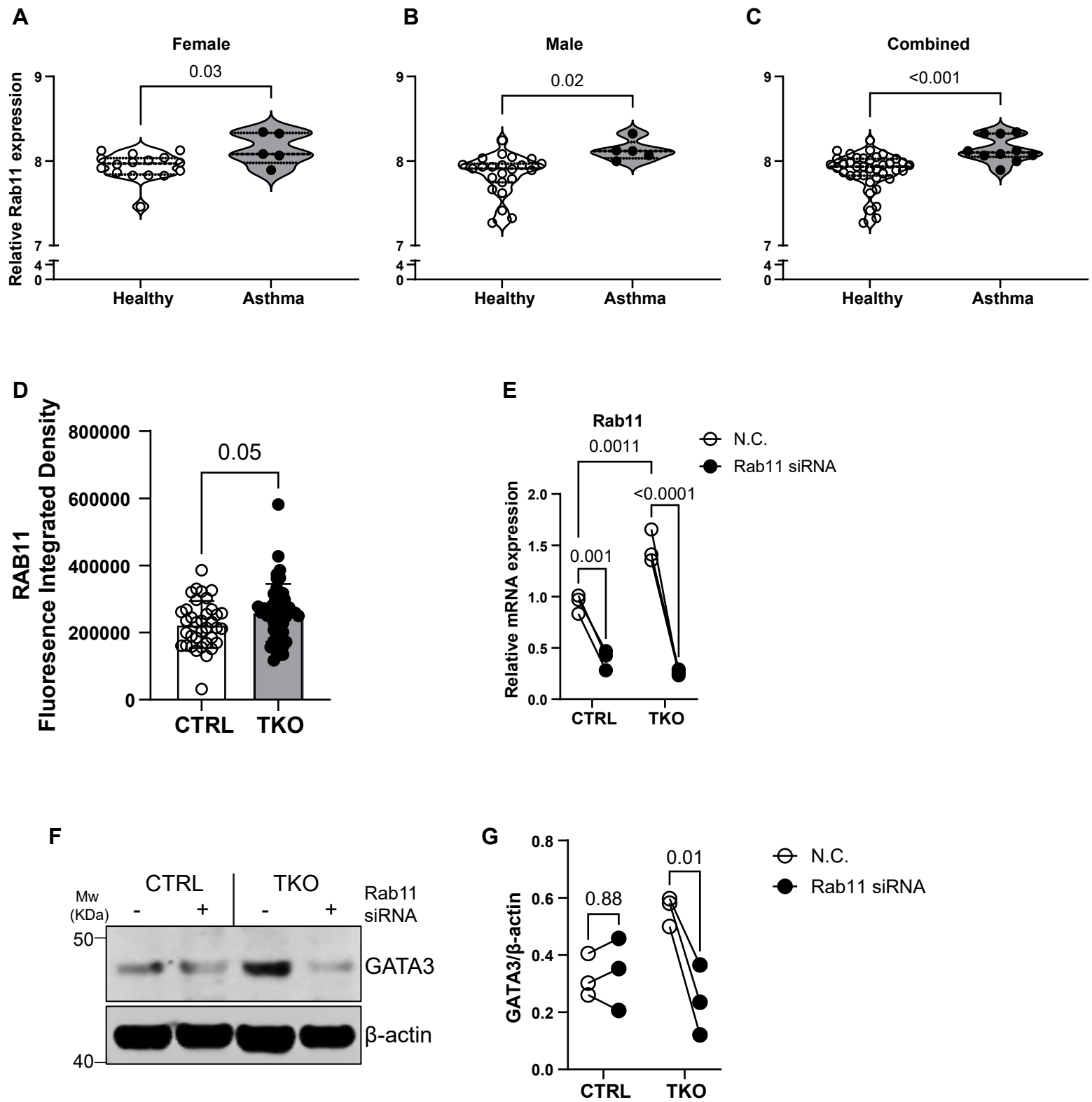

Supplement: Supplement 2 — Supplementary Figure 4. Increased RAB11 expression in asthma and functional effects of RAB11 depletion in CD4+ T cells. (A-C) Analysis of publicly available transcriptomic datasets (GSE123086) showing increased RAB11 expression in CD4+ T cells from female asthma patients (A), male asthma patients (B), and combined cohorts (C) compared with healthy controls. (D) Quantification of RAB11 fluorescence integrated density from confocal microscopy images of CTRL and TKO CD4+ T cells. (E) Quantification of Rab11 mRNA expression following siRNA-mediated knockdown in CTRL and TKO CD4+ T cells confirming efficient RAB11 depletion. (F) Immunoblot analysis of GATA3 levels in CTRL and TKO CD4+ T cells following transfection with control or Rab11 siRNA. β-actin was used as a loading control. (G) Densitometric quantification of GATA3 protein levels normalized to β-actin. Data are presented as mean ± SEM. Statistical analysis was performed using an unpaired two-tailed Student’s t-test. [file media-2.pdf]

Supplementary Figure 5

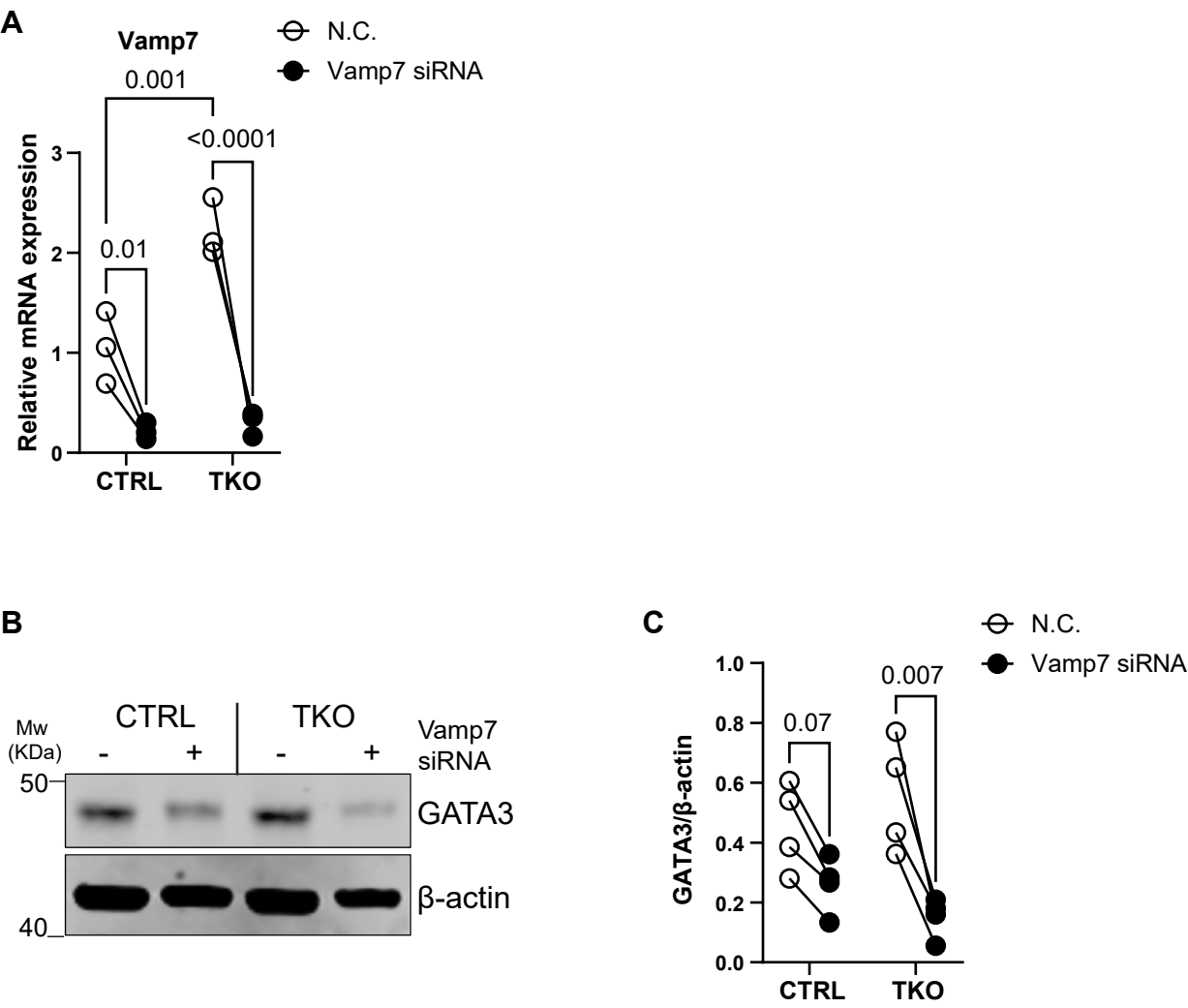

Supplement: Supplement 3 — Supplementary Figure 5. Efficient VAMP7 depletion reduces GATA3 expression in CD4+ T cells. (A) Quantification of Vamp7 mRNA expression following siRNA-mediated knockdown in CTRL and TKO CD4+ T cells. (B) Immunoblot analysis of GATA3 expression in CTRL and TKO CD4+ T cells transfected with control or Vamp7 siRNA. β-actin was used as a loading control. (C) Densitometric quantification of GATA3 protein levels normalized to β-actin in CTRL and TKO CD4+ T cells. Data are presented as mean ± SEM. Statistical analysis was performed using an unpaired two-tailed Student’s t-test. [file media-3.pdf]
